# Revised placement of *Epilandex* Hebard, 1927 in Anisolabididae (Insecta, Dermaptera) with a re-evaluation of *Carcinophora kawakamii* (Shiraki, 1928) and *Epilandex acuminata* Srivastava, 2003

**DOI:** 10.3897/zookeys.1294.201081

**Published:** 2026-10-06

**Authors:** Meliana Gusti, Yoshitaka Kamimura, Bounsanong Chouangthavy, Chi-Feng Lee, Muhammad Silmi, Ali Amran I, Katsuyuki Eguchi

**Affiliations:** 1 Department of Biological Sciences, Graduate School of Science, Tokyo Metropolitan University, Tokyo, 192-0397, Japan Department of Biological Sciences, Graduate School of Science, Tokyo Metropolitan University Tokyo Japan; 2 Biodiversity of Sumatra Research Group, Universitas Negeri Padang, West Sumatra, 25131, Indonesia Biodiversity of Sumatra Research Group, Universitas Negeri Padang West Sumatra Indonesia; 3 Department of Biology, Keio University, Kanagawa, 223-8521, Japan Department of Biology, Keio University Kanagawa Japan; 4 Entomology Laboratory, Faculty of Agriculture, National University of Laos, Vientiane, Laos Entomology Laboratory, Faculty of Agriculture, National University of Laos Vientiane Laos; 5 Zoology Division, Taiwan Agricultural Research Institute, Taichung, Taiwan Zoology Division, Taiwan Agricultural Research Institute Taichung Taiwan; 6 Biodiversity Division, United Plantations Berhad / PT Surya Sawit Sejati, Pangkalan Bun, Indonesia Biodiversity Division, United Plantations Berhad / PT Surya Sawit Sejati Pangkalan Bun Indonesia

**Keywords:** Earwigs, *Epilandex kawakamii* comb. nov., Epilandicinae nom. nov., male genitalia, molecular phylogeny, spermatheca

## Abstract

The earwig genus *Epilandex* Hebard, 1927 (type species: *Landex burri* Borelli, 1921) consists of 14 named, small anisolabidid earwig species known from both the Old and New Worlds, and is characterized by long, acuminate parameres in the male genitalia. This study generated the first molecular data for newly obtained *Epilandex* specimens and evaluated its phylogenetic position within Anisolabididae. We analyzed combined mitochondrial and nuclear gene data, in conjunction with a re-examination of their external and genital morphology. Although this genus has traditionally been placed in Anisolabidinae, our results demonstrate that *Epilandex* should be recognized as a separate clade from other members of this subfamily. The name “Landicinae,” which had been used for such a treatment, is a synonym of Anisolabidinae, due to the misidentification of the type genus, and therefore we propose the new name Epilandicinae. Furthermore, based also on the examination of museum specimens, this study revises species delineations within *Epilandex*, including the proposal of *Ep. kawakamii***comb. nov**. The female genitalia of this genus is also reported for the first time, together with new distribution records of two species in Asia.

## Introduction

The order Dermaptera comprises more than 2,000 extant species (Popham 2000; Grimaldi and Engel 2005; Haas 2018; Hopkins et al. 2025), distributed across a variety of natural and semi-natural habitats (Haas 2018). The phylogeny of Dermaptera has been investigated in several studies (Haas 1995; Colgan et al. 2003; Jarvis et al. 2005; Wipfler et al. 2020; Kočárek et al. 2024). According to the recent proposal by Kamimura (2025), extant dermapteran species (Neodermaptera) are divided into three major groups based on the number and orientation of the penis lobe(s): those with paired penis lobes oriented proximally at the time of emergence (Infraorder Protodermaptera); those with paired penis lobes oriented distally at the time of emergence (Infraorder Epidermaptera: Parvorder Mesodermaptera); and those with the penis lobe reduced to a single, distally oriented one (Epidermaptera: Parvorder Eudermaptera).

Within the parvorder Mesodermaptera, the family Anisolabididae comprises 11 subfamilies, including Anisolabidinae (Srivastava 1999; Haas 2018; Kamimura 2025). Due to the high species richness of the subfamily Anisolabidinae with approximately 300 species (Anisyutkin 1998, 2004; Srivastava 1999, 2003a, 2003b; Nishikawa 2008; Kočárek 2021; Nishikawa and Jaitrong 2023), its internal classification is particularly problematic. Moreover, the relationships among the subfamilies of Anisolabididae remain largely unresolved, primarily due to biased taxon sampling focused on only a limited number of species of Anisolabidinae (Haas 2018; Kočárek et al. 2024). Consequently, there is no consensus regarding synapomorphic characters to define each subfamily of Anisolabididae.

The genera within the subfamily have been defined primarily based on the shape of a single male genital structure, the parameres (= external parameres or metaparameres of Steinmann 1986), resulting in severe confusion and inconsistencies among researchers (Srivastava 2003a; Kočárek 2014; Anisyutkin 2020). The lack of distinct morphological differences among internal taxa has posed a significant challenge in determining generic boundaries (Anisyutkin and Thinh 2022). According to Hopkins et al. (2025), Anisolabidinae consists of 19 valid genera, some of which are widespread in Southeast Asia (Hebard 1927; Anisyutkin 1998; Srivastava 1999; Nishikawa and Jaitrong 2023). Despite their wide distribution, the relationships among genera within this subfamily have not been fully resolved.

During our comprehensive study addressing this gap, which focuses mainly on the fauna and phylogeny of the anisolabidid species from Indonesia and adjacent Southeast Asian countries, the first author obtained specimens of the genus *Epilandex* Hebard, 1927 from Taiwan, Laos, and Borneo (Kalimantan). This study aims to identify the species represented by these specimens based on both morphological and molecular evidence. We also analyzed the phylogenetic position of *Epilandex* within Anisolabididae by reconstructing a molecular phylogenetic tree incorporating newly obtained DNA sequences of the genus. Furthermore, the spermatheca of the genus *Epilandex* is described for the first time, together with updated distribution records.

## Material and methods

### Material examined and observations

A total of 24 adult specimens of anisolabidid species (Anisolabidinae and Brachylabidinae), collected during various field surveys, were examined. The specimens obtained in the field were immediately preserved in 90% or higher ethanol. The external morphology was examined under a SMZ1270 stereomicroscope (Nikon, Tokyo, Japan). For comparative observation of the metasternal morphology, we also comprehensively reviewed records reported in previous studies, primarily those in Sakai (1987).

Genital structures were examined following the methods of Kamimura et al. (2023). After softening the abdomen in hot water, the genitalia were removed, cleared in a 10% KOH solution, and mounted in Euparal between two coverslips. A Nikon Eclipse E600 microscope was used to observe genital details and take photographs. The terminology used for female and male genital structures largely follows that of Schneider and Klass (2013) and Kamimura (2025), respectively.

Male body parts and genitalia were measured using ImageJ 1.49m (National Institutes of Health, USA, available at http://imageJ.nih.gov/ij/) based on images captured with an EOS R10 digital camera (Canon, Tokyo, Japan) attached to a Nikon AZ100 stereomicroscope. Focus-stacked images were produced using Helicon Focus Pro (Helicon Soft Ltd., http://www.heliconsoft.com/) from a series of source images captured with the aforementioned imaging system. Artifacts, ghosting, and extraneous elements, such as out-of-focus appendages and tissue, surrounding or covering the target objects were removed using the retouching function of Helicon Focus Pro. Color balance, contrast, and sharpness were adjusted using Adobe Photoshop CS6.

Examination of type specimens was conducted using high-resolution photographs provided by the curators of the respective institutions. Photographs of the holotype of *Psalis kawakamii* Shiraki, 1928, a species that many contemporary researchers place in *Carcinophora* Scudder, 1876, and the holotype and allotype of *Epilandex peterseni* Ramamurthi, 1967, were provided by the National Taiwan University Museum and the Natural History Museum of Denmark (University of Copenhagen), respectively. These images included the dorsal, ventral, and lateral views used for morphological comparison. For the identification of the examined *Epilandex* specimens, we referred to previous studies by Shiraki (1928), Ramamurthi (1967), and Srivastava (1999), as well as the most recent study by Kočárek (2021).

### Molecular methods

DNA from a total of 26 anisolabidid specimens, along with three sequences representing two species from GenBank (Table 1), was analyzed to determine their phylogenetic placement. Two species of Labiduridae, a family considered the sister group to Anisolabididae in many previous studies (Kamimura 2025), were selected as outgroups. Following previous studies (Giribet et al. 1996; Colgan et al. 2003; Jarvis et al. 2005; Kočárek et al. 2024), phylogenetic analyses were performed using partial sequences of three genetic markers: the cytochrome *c* oxidase subunit I gene (COI, 663 bp: the universal DNA barcode region), 18S ribosomal DNA (18S, 714 bp), and 28S ribosomal DNA (28S, 363 bp) (Table 2). The genetic and phylogenetic relationships among *Epilandex* specimens from multiple localities were also analyzed based on the COI barcode region.

**Table 1.** Details of the specimens used in the phylogenetic analyses. Abbreviations: N/A-not available.

**Subfamily**	**Species**	**Specimens ID/ References**	**COI**	**18S**	**28S**	**Sex**	**Locations**
**Anisolabidinae Verhoeff, 1902**	*Anisolabis maritima*	27vi25-3J	PZ403161	PZ405228	PZ406483	Male	Japan
		21v25-4J	PZ403160	PZ405223	PZ406482	Male	Japan
	*Anisolabis picea*	8viii25-9J	PZ405780	PZ405229	PZ406484	Female	Japan
		8viii25-10J	PZ405781	PZ405230	PZ406485	Male	Japan
	*Anisolabella marginalis*	26v25-2J	PZ405778	PZ405224	PZ406481	Male	Japan
		12xi25-15J	PZ405779	N/A	N/A	Male	Japan
	*Anisolabella ryukyuensis*	21xi22-7YK	PZ403157	N/A	N/A	Male	Japan
	*Gonolabis electa*	6xii23-1GP	PZ403158	PZ405227	PZ406479	Male	Indonesia: Sumatra
		6xii23-4GP	PZ403159	PZ405225	PZ406480	Male	Indonesia: Sumatra
	*Euborellia annulata*	24i23-1IR	PZ394338	N/A	PZ406478	Female	Indonesia: Sumatra
	*Euborellia pallipes*	14vi22-3YK	PZ403156	N/A	N/A	Male	Japan
	*Euborellia plebeja*	Kalaentzis et al. 2021	W703670	N/A	N/A	Male	Thailand
	*Epilandex peterseni*	15iii24-1BN	PZ405769	PZ405218	PZ406488	Male	Indonesia: Borneo
		15iii24-3BN	PZ405770	N/A	N/A	Male	Indonesia: Borneo
		15iii24-4BN	PZ405771	PZ405221	PZ406489	Female	Indonesia: Borneo
		15iii24-6BN	PZ405768	N/A	N/A	Female	Indonesia: Borneo
	*Epilandex kawakamii* comb. nov.	13vii23-1TW	PZ405777	N/A	N/A	Male	Taiwan
		13vii23-3TW	PZ405772	N/A	N/A	Male	Taiwan
	*Epilandex kawakamii* comb. nov.	25viii24-11LS	PZ405773	PZ405220	PZ406487	Male	Laos
		25viii24-12LS	PZ405775	PZ405219	PZ406486	Male	Laos
		25viii24-15LS	PZ405774	N/A	N/A	Male	Laos
		25viii24-16LS	PZ405776	N/A	N/A	Male	Laos
**Brachylabidinae Burr, 1908**	*Metisolabis* sp. 1	18vii24-4AL	PZ405782	N/A	PZ406476	Female	Indonesia: Sumatra
	*Metisolabis* sp. 2	28vi22-31LS	PZ405783	PZ405226	PZ406477	Female	Laos
**Outgroup (Labiduridae Verhoeff, 1902)**	*Labidura riparia*	27xii24-17TW	PZ403155	PZ405222	PZ406490	Female	Taiwan
	*Nala ornata*	Kočárek et al. 2024	OQ799520	OQ780436	N/A	Male	Brunei

**Table 2.** Primer used in Amplification and sequencing.

**Gene**	**Primer name**	**Sequences**	**References**
COI	LCO1490	GGTCAACAAATCATAAAGATATTGG	Folmer et al. 1994
	HCO2198	TAAACTTCAGGGTGACCAAAAAATCA	–
28SrDNA	D1F	ACCCSCTGAAYTTAAGCAT	Hillis and Dixon 1991
	D1R	AACTCTCTCMTTCARAGTTC	–
18SrDNA	1F	TACCTGGTTGATCCTGCCAGTAG	Giribet et al. 1996
	5R	CTTGGCAAATGCTTTCGC	–

After each specimen was washed in 500 μL of TE buffer (pH 8.0) in a sterile disposable plate, a leg was removed and transferred to a tube containing 105 μL of DNA extraction solution (100 μL of Chelex-TE solution and 5 μL of Qiagen proteinase K; Satria et al. 2015). This mixture was incubated at 56 °C for 24 h and then heated at 99 °C for 10 min to inactivate the enzyme. Each PCR reaction consisted of 5 μL of 2× PCR Buffer, 2 μL of 2 mM dNTPs, 0.3 μL of forward primer (10 μM), 0.3 μL of reverse primer (10 μM), 1.7 μL of distilled water, 0.2 μL of KOD FX Neo DNA polymerase (1.0 unit/μL; Toyobo, Osaka, Japan), and 1 μL of DNA template. PCR amplifications were performed using a MiniAmp Thermal Cycler (Thermo Fisher Scientific, Waltham, Massachusetts, USA) under the following conditions: for COI, 94 °C for 2 min, followed by 35 cycles of 94 °C for 15 s, 51 °C for 15 s, and 72 °C for 15 s, with a final extension at 72 °C for 6 min; for 18S, 95 °C for 5 min, followed by 35 cycles of 95 °C for 45 s, 50 °C for 45 s, and 72 °C for 1 min, with a final extension at 72 °C for 5 min; and for 28S, 95 °C for 4 min, followed by 35 cycles of 95 °C for 30 s, 52 °C for 1 min, and 72 °C for 1 min, with a final extension at 72 °C for 5 min. All reaction products were held at 4 °C. Amplified fragments were verified on a 2.4% agarose gel. For ExoSAP treatment, 2 μL of diluted ExoSAP solution (0.1 μL of ExoSAP and 1.9 μL of distilled water) was added to 5 μL of PCR product, followed by incubation at 37 °C for 4 min and 80 °C for 1 min, and finally held at 4 °C. The ExoSAP-treated products were then sequenced by FASMAC (Kanagawa, Japan). The chromatograms were manually examined and assembled using ChromasPro v. 1.7.6 (Technelysium Pty Ltd., Brisbane, Queensland, Australia).

### Phylogenetic analyses

The cleared sequences were aligned using MUSCLE v. 3.8.425 (Edgar 2004) implemented in AliView v. 1.28 (Larsson 2014). The multigene datasets were then concatenated using Mesquite v. 3.81. The concatenated dataset was partitioned by gene and codon position. The best-fitting substitution models for each partition were selected using the automatic function provided by the IQ-TREE v. 3.0.1 software under a Bayesian Information Criteria (BIC).

Phylogenetic trees were reconstructed using maximum likelihood (ML) and Bayesian inference (BI) methods. The ML analysis was performed using IQ-TREE (Nguyen et al. 2015; Minh et al. 2020), with the following models selected: TN+F+I, F81+F+I, HKY+F+R2 for the first, second, and third positions of COI, respectively; F81+I for 18S; and TPM3u+F+I for 28S. Node support was assessed using ultrafast bootstrap with 1,000 replicates (Minh et al. 2013). The BI was conducted using MrBayes v. 3.3.7a with flat priors via the CIPRES Science Gateway v. 3.3. using a Markov chain Monte Carlo (MCMC) method. In addition, for the BI analysis, the selected models were translated into the corresponding models available in MrBayes and applied to the respective partitions. Two independent MCMC runs, each consisting of four chains, were run for 10,000,000 generations with a temperature parameter of 0.2. Trees and parameters were sampled every 1,000 generations, and the first 25% of the sampled values were discarded as burn-in. The final average standard deviation of split frequencies (ASDSF) value was 0.002640, with an average potential scale reduction factor (PSRF) of 1.000 and a maximum PSRF of 1.001. The phylogenetic trees were visualized and edited using FigTree v. 1.4.4 (Drummond and Rambaut 2007).

To assess genetic variation, pairwise genetic distances (*p*-distance) among *Epilandex* species across sampling localities were calculated using MEGA v. 12.0.10.

## Results

### Molecular analysis

**Figure 1.**
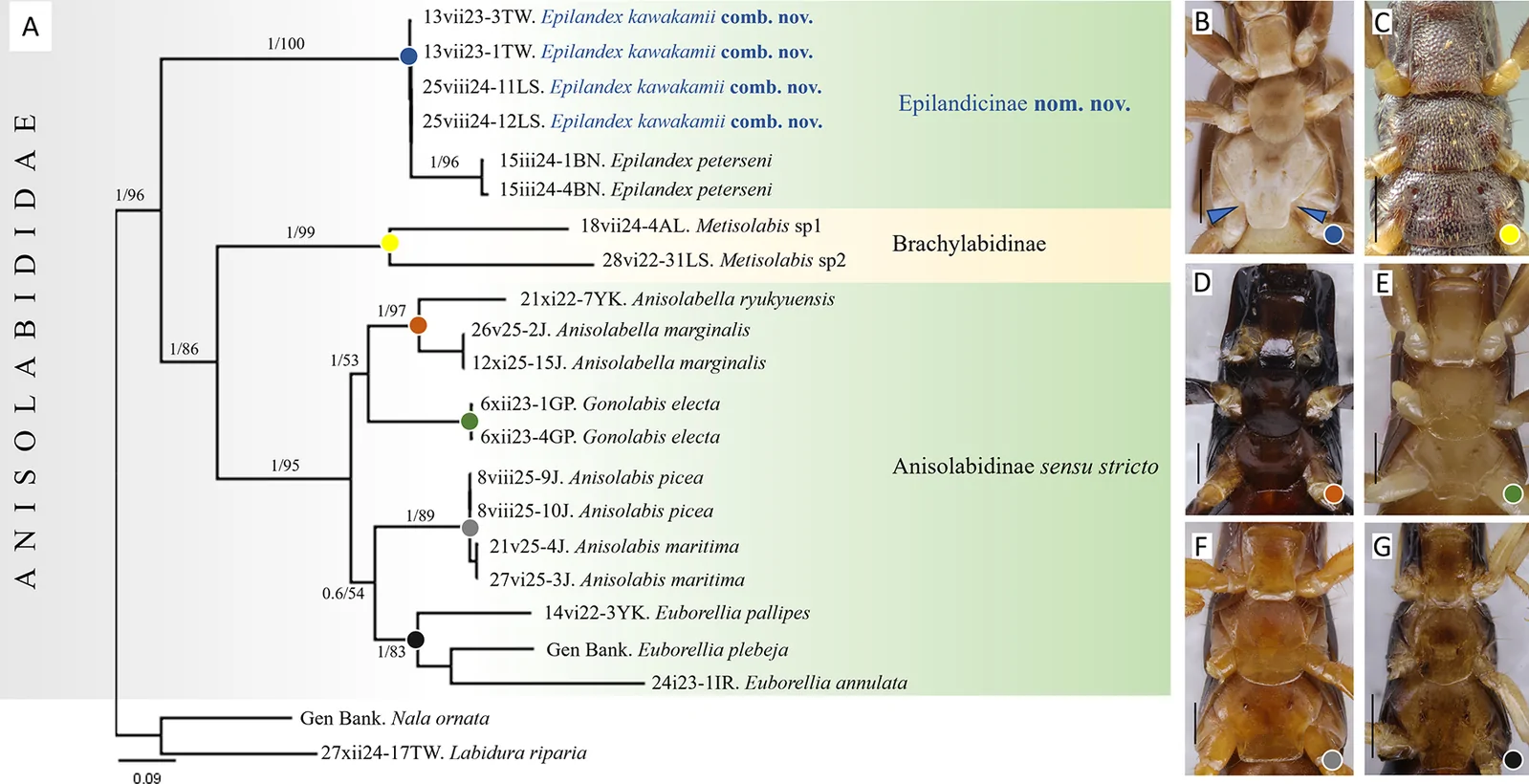
The combination of molecular data and morphology. **A**. Maximum likelihood tree based on the combined dataset (COI, 18S, and 28S). Bayesian posterior probabilities (PPs) and IQ-TREE bootstrap support values (BS) are shown for each branch with BS > 50. The new taxon names proposed in this study are shown in blue; **B–G**. Metasternum of the representative species of the six clade (indicated by circles of different colors; **B**. 13vii23-1TW; **C**. 18vii24-4AL; **D**. 12xi25-15J; **E**. 6xii23-1GP; **F**. 21v25-4J; **G**. 14vi22-3YK). Blue arrowheads in (**B**) indicate the conspicuous constriction of the metasternum in *Epilandex* species. Scale bars: 1 mm.

Of the 24 anisolabidid specimens examined in this study, ten were identified as belonging to the genus *Epilandex* based on the morphological diagnoses proposed in previous studies (Shiraki 1928; Ramamurthi 1967; Srivastava 1999; Kočárek 2021). In the phylogenetic tree reconstructed from combined mitochondrial and nuclear genes, the representatives of four genera of Anisolabidinae (*Anisolabis* Fieber, 1853; *Anisolabella* Zacher, 1911; *Euborellia* Burr, 1909; and *Gonolabis* Burr, 1900) formed a clade. However, the *Epilandex* specimens were clearly separated from this clade. This placement was strongly supported by high bootstrap values and posterior probabilities, positioning Brachylabidinae as the sister group to Anisolabidinae excluding *Epilandex* (i.e., Anisolabidinae*sensu stricto*) (Fig. 1A).

**Figure 2.**
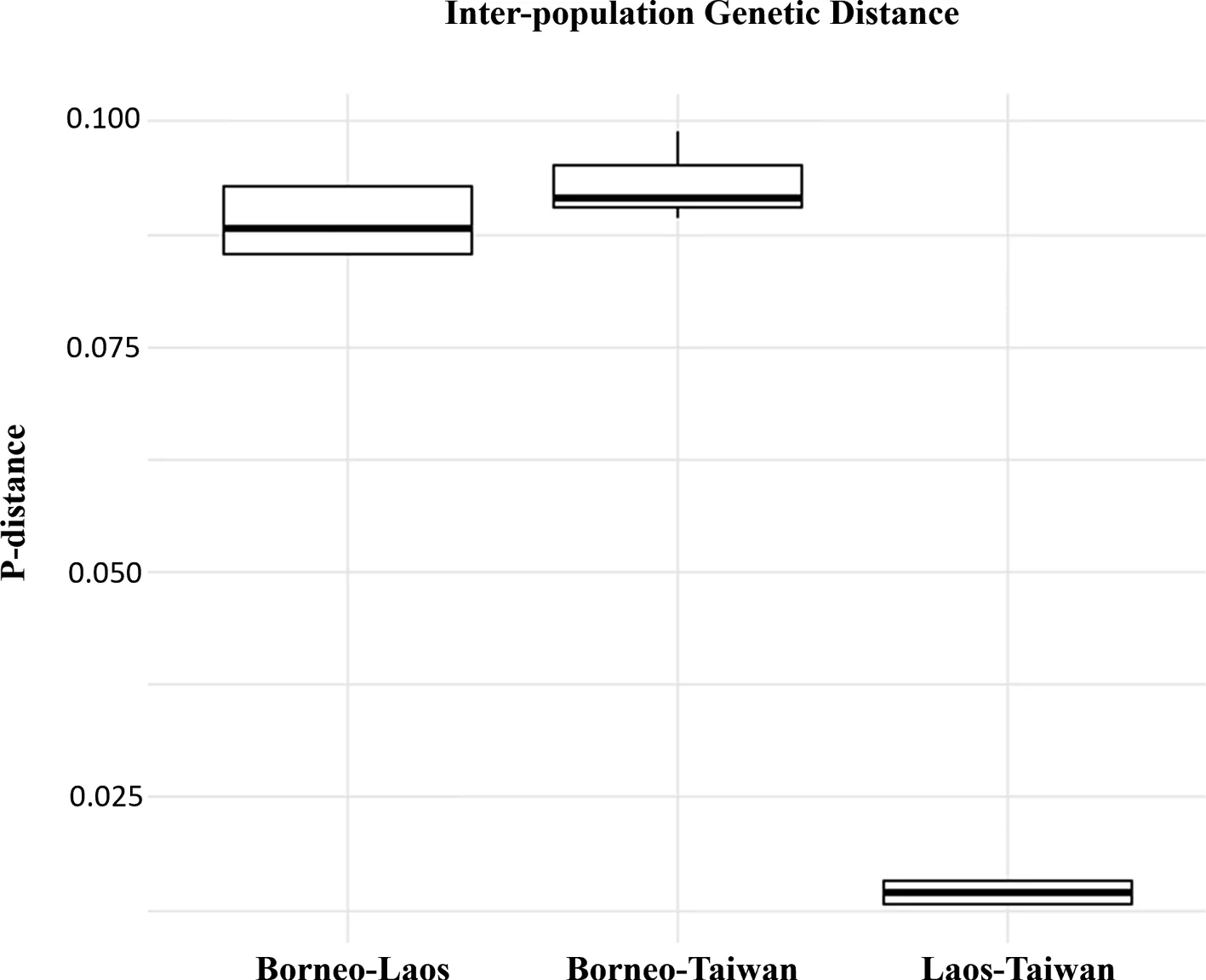
Genetic distances among populations: Box plots show the distribution of pairwise genetic distances for each population comparison (Borneo–Laos, Borneo–Taiwan, and Laos–Taiwan). The central line represents the median, the boxes represent the interquartile range, and the whiskers represent the range of the data.

In the phylogenetic tree, the *Epilandex* specimens from Borneo formed a distinct clade nested among the specimens from Taiwan and Laos (Fig. 1A). These two groups were clearly separated into two distinct clades in our COI-only analysis, which included additional specimens (Suppl. material 1). The specimens from Taiwan and Laos formed a clade with a very low degree of internal divergence (maximum *p*-distance of 1.56%; Fig. 2; Suppl. material 1). The specimens from Borneo formed another clade with a similarly low degree of internal divergence (2.12%), but exhibited more than 9% divergence from the Taiwan+Laos clade (Fig. 2), indicating that they are heterospecific.

### Morphological examination

**Figure 3.**
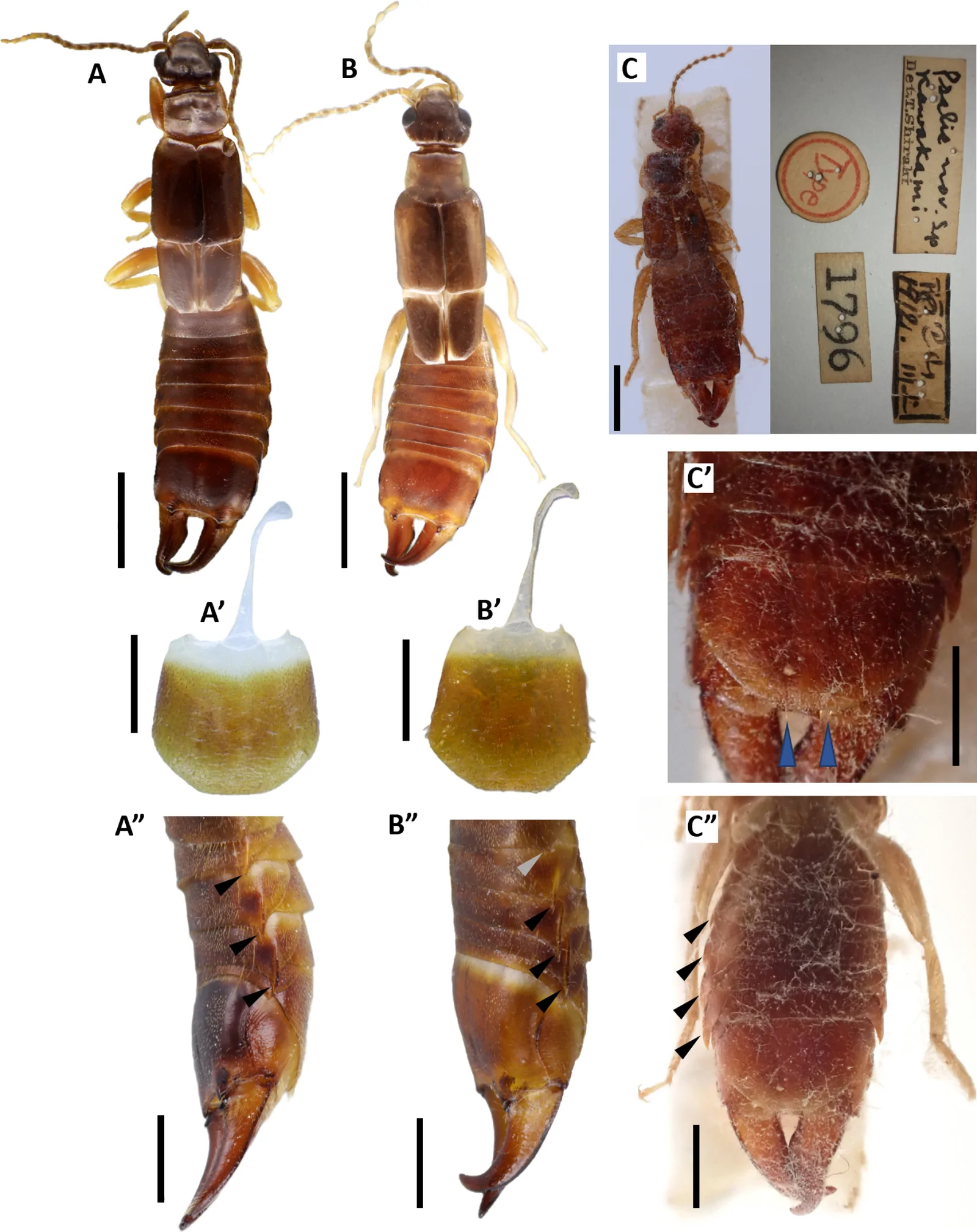
Habitus of *Epilandex kawakamii* comb. nov. **A**–**A”**. Male specimen from Taiwan (13vii23-1TW); **B**–**B’’**. Male specimen from Laos (25viii24-11LS); **C**–**C”**. Holotype of *Psalis kawakamii* (male). Habitus, penultimate sternite (**A’–C’**), lateral view on abdomen (**A”–B”**), and ventral view on abdomen (**C”**) are shown. Blue arrowheads in (**C’**) indicate the prominent tip of the parameres covered by the penultimate sternite. Black arrowheads in (**A”–C”**) indicate well-developed median longitudinal carina. Grey arrowhead in (**B’’**) indicates trace of carina with a spine. Scale bars: 2 mm (**A–C**); 1 mm (**A’–C”**).

**Figure 4.**
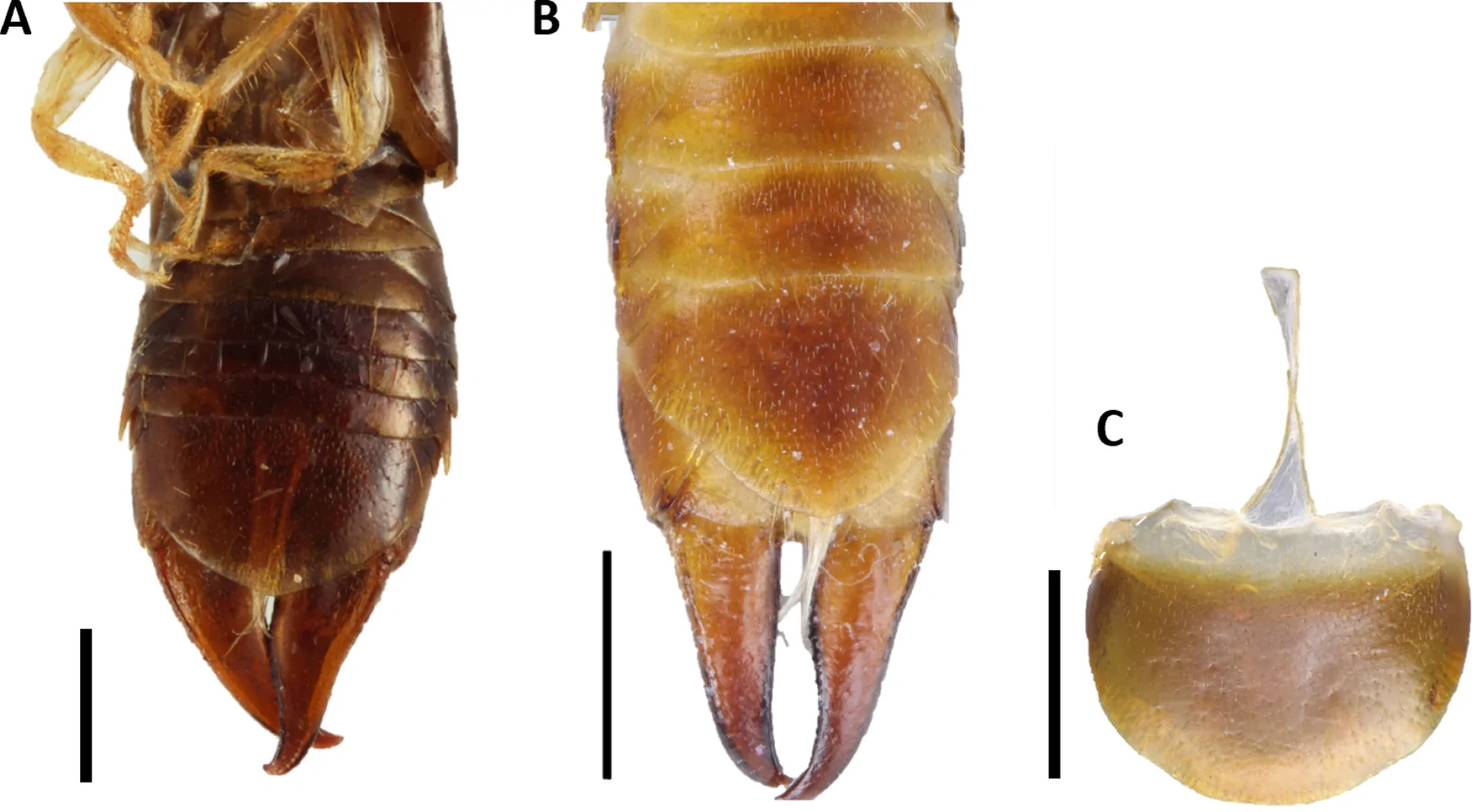
*Epilandex peterseni*. **A**. Ventral view on abdomen of holotype; **B–C**. Ventral view on abdomen (**B**) and penultimate sternite (**C**) of a specimen from Borneo (15iii24-1BN). Scale bars: 1 mm.

The newly examined *Epilandex* specimens exhibited similar external and genital morphology, including a small body size, carinae on each side of several abdominal tergites, long parameres, long virgae with a basal appendage, and a long tubular spermatheca (Figs 3A, 3A”, 3B, B”, 4, 5). However, they differed in the shape of the posterior margin of the penultimate sternite, which was almost truncate or slightly emarginate medially in the Taiwan and Laos populations (Fig. 3A’, B’), but distinctly rounded in the Bornean clade (Fig. 4C). Furthermore, no conspicuous morphological differences were detected between the Taiwanese and Laotian populations. Consequently, the integrative taxonomic approach employed in this study consistently supports the existence of two distinct species among the examined *Epilandex* specimens.

Our examination revealed that *Epilandex* consistently possesses a metasternum that narrows posteriorly, with a distinct constriction at the base of the hind legs (Fig. 1B). In contrast, in Anisolabidinae excluding *Epilandex* (= Anisolabidinae*sensu stricto*) (Fig. 1A), the metasternum narrows posteriorly without such a constriction (Fig. 1D–G). This latter condition was also found in the clade of Brachylabidinae (Fig. 1C). Our literature review further confirmed this difference between *Epilandex* and the clade comprising Anisolabidinae*sensu stricto* and Brachylabidinae.

### Morphology of museum materials

Our examination of the holotype (male) of *Epilandex peterseni* confirmed its rounded penultimate sternite and long parameres that protrude conspicuously beyond the posterior margin of the penultimate sternite (Fig. 4A). This holotype also closely matches the Bornean specimens examined in this study in other morphological respects. Although we could not examine the type specimens of *Ep. acuminata* Srivastava, 2003, a species known only from its type locality (Hong Kong) to date, its morphology is highly similar to that of *Ep. peterseni*, except for the medially emarginate posterior margin of the penultimate sternite (Srivastava 2003a; Kočárek 2021).

Our examination of the holotype (male) of *Psalis kawakamii* (= *Carcinophora kawakamii*), a species known from Taiwan (type locality) and China (Fujian), revealed that it exhibits external morphology consistent with the genus *Epilandex* (Fig. 3C, C’’). Specifically, we observed the tips of the long parameres protruding beyond the penultimate sternite, which is medially emarginate (Fig. 3C’). Based on the evidence presented above, we propose *Epilandex acuminata* as a junior synonym of *Epilandex kawakamii* comb. nov., and provide additional and new distribution records from Taiwan and Laos, respectively.

### Taxonomy and nomenclature

#### Family Anisolabididae Verhoeff, 1902

##### Subfamily Epilandicinae

Taxon classificationAnimaliaDermapteraAnisolabididae

Gusti, Kamimura & Eguchi nom. nov.

C0A83C1A-C939-5AE0-973A-7FE52965E804

###### Type genus.

*Epilandex* Hebard, 1927.

The genus *Epilandex* presently consists of 14 described species: nine species from the Old World (Oriental and Australasian) (Kočárek 2021) and five species from Central and South America (formerly placed in *Capralabis*Brindle 1981 but transferred to *Epilandex* by Srivastava (1999)).

The genus *Landex* was established by Burr (1915) for *Labidura femoralis* Dohrn, 1863, and was characterized by long and narrow parameres and asymmetrical forceps. For this monotypic genus, Burr (1915) also proposed the subfamily Landicinae. Later, Borelli (1921) described *Landex burri* based on the type specimens from Bangkok, Siam (= Thailand), with diagnostic characters that closely corresponded to the definition of the genus *Landex* given by Burr (1915). However, in the same publication, he also noted that the material examined by Burr (1915) consisted of two clearly distinct species, one of which was *Labidura femoralis* (as *Psalis femoralis*), but he did not explain how the name *Landex* should be restricted. Many contemporary studies place *Labidura femoralis* in *Euborellia*, because of its square-shaped parameres (Steinmann 1986; Srivastava 1990, 2003a; Anisyutkin 1998). Thus, the misidentification in Burr (1915) resulted in an inconsistency between the definitive statements of *Landex* and the type species (*Labidura femoralis*). To resolve this situation, Hebard (1927) established the genus *Epilandex* under Psalinae (= Anisolabidinae), with *Landex burri* Borelli, 1921 designated as its type species. Later, Boeseman (1954) placed the genus *Epilandex* in the subfamily Landicinae. However, this treatment is problematic because he simultaneously placed the type species of *Landex* (as *Psalis femoralis*) within a distinct subfamily Psalinae. Finally, Engel and Haas (2007) synonymized Landicinae with Anisolabidinae.

###### Diagnosis.

(modified from Burr 1915) Monotypic subfamily comprising only the type genus. Small earwigs resembling members of the subfamily Anisolabidinae. Eyes large, almost as long as or longer than postocular length. Third antennal segment long and cylindrical, 4^th^ and onwards pyriform. Tegmina well-developed (except for *Ep. wagneri*); hindwings developed, reduced or wanting; prosternum subparallel, weakly constricted posteriorly; mesosternum rounded posteriorly; metasternum notably constricted posteriorly at the base of hind coxae; legs not long; tarsi slender, third segment ~ 1/2 as long as the first. Abdomen not depressed, parallel-sided; each side with a developed carina posteriorly in males of some species; forceps short and stout, generally asymmetrical in males; manubrium parallel-sided, rectangular, ~ 2× as long as broad. Male genitalia with laterally paired penis lobes pointing in opposite directions when in repose; basal piece long and slender; parameres very long and narrow, length at least 6 × (usually > 10) width at widest basal part; virga very long and slender; basal vesicle with or without a sclerotized plate.

### Key to the subfamilies of Anisolabididae modified based on Srivastava (1999) (on males only)

**Table:** 

1	Body strongly flattened, i.e., dorsoventrally compressed	**Platylabiinae Burr, 1911**
–	Body not strongly flattened, head and abdominal tergites often convex or weakly convex or flattened	**2**
2	Metasternum with hind margin convex, more or less tongue shaped	**Titanolabidinae Srivastava, 1982**
–	Metasternum with hind margin truncate. In some species rounded but the region narrowed between hind coxae not elongate, not tongue shaped	**3**
3	Mesosternum with hind margin convex	**4**
–	Mesosternum with hind margin truncate. Even when the hind margin is broadly rounded, it remains transverse overall and does not form a semicircular posterior portion	**6**
4	Eyes absent, parameres vestigial or reduced	**Anophthalmolabidinae Steinmann, 1975**
–	Eyes and parameres well developed	**5**
5	Parameres conspicuously elongate; length at least 6× width at widest basal part, often > 10 ×; apex sharply pointed. Metasternum narrowed posteriorly, with constriction at the base of hind legs	**Epilandicinae nom. nov**.
–	Paramere length 5× or less the width at widest part. Metasternum narrowed posteriorly, without any constriction at the base of hind legs	**Anisolabidinae Verhoeff, 1902**
6	First antennal segment longer than the distance between antennal bases	**7**
–	First antennal segment shorter or approx. as long as the distance between antennal bases	**8**
7	Mesonotum laterally with a sharp ridge or convexity along its whole length, often dying out in posterior half	**Brachylabidinae Burr, 1908**
–	Mesonotum laterally without a ridge or convexity	**Isolabidinae Verhoeff, 1902**
8	Basal piece of central parameral plate not deeply incised posteriorly	**Antisolabidinae Brindle, 1978**
–	Basal piece of central parameral plate deeply incised posteriorly, largely divided into two parts	**9**
9	Penis lobe of both sides equally developed	**10**
–	Right penis lobe atrophied	**Isolaboidinae Brindle, 1978**
10	Ultimate tergite narrowed posteriorly, setose (*Parisolabis*: endemic to New Zealand) or not setose (*Parisopsalis*: endemic to Australia)	**Parisolabidinae Verhoeff, 1904**
–	Ultimate tergite not setose, lateral sides almost parallel. Known from Central and South America (*Idolopsalis*)	**Idolopsalinae Steinmann, 1975**

#### Genus Epilandex

Taxon classificationAnimaliaDermapteraAnisolabididae

Hebard, 1927

2480A7E3-E281-5A93-B919-7FE433B52AF2

##### Type species.

*Landex burri* Borelli, 1921.

##### List of species.

Modified from Srivastava 1999; Kočárek 2021:

*Epilandex ashmolei* (Brindle, 1981)

(= *Capralabis ashmolei* Brindle, 1981)

*Epilandex borellii* Srivastava, 1999

(= *Capralabis burri* (Borelli, 1905))

*Epilandex burri* (Borelli, 1921)

*Epilandex gressitti* Srivastava, 1976

*Epilandex handschini* Hincks, 1954

*Epilandex kawakamii* (Shiraki, 1928), comb. nov.

(= *Epilandex acuminata* Srivastava, 2003, syn. nov.)

*Epilandex nigra* Caudell, 1907

(= *Capralabis nigra* (Caudell, 1907))

*Epilandex peterseni* Ramamurthi, 1967

*Epilandex serrata* Srivastava, 1976

*Epilandex solomonensis* Brindle, 1970

*Epilandex srivastavai* (Brindle, 1981)

(= *Capralabis srivastavai* Brindle, 1981)

*Epilandex steinmanni* (Brindle, 1981)

(= *Capralabis steinmanni* Brindle, 1981)

*Epilandex wagneri* Srivastava, 1990

*Epilandex vignai* Kočárek, 2021

#### Epilandex kawakamii

Taxon classificationAnimaliaDermapteraAnisolabididae

(Shiraki, 1928) comb. nov.

7E87F567-EFA7-5C6D-92C1-DE21369042CC

**Figure 5.**
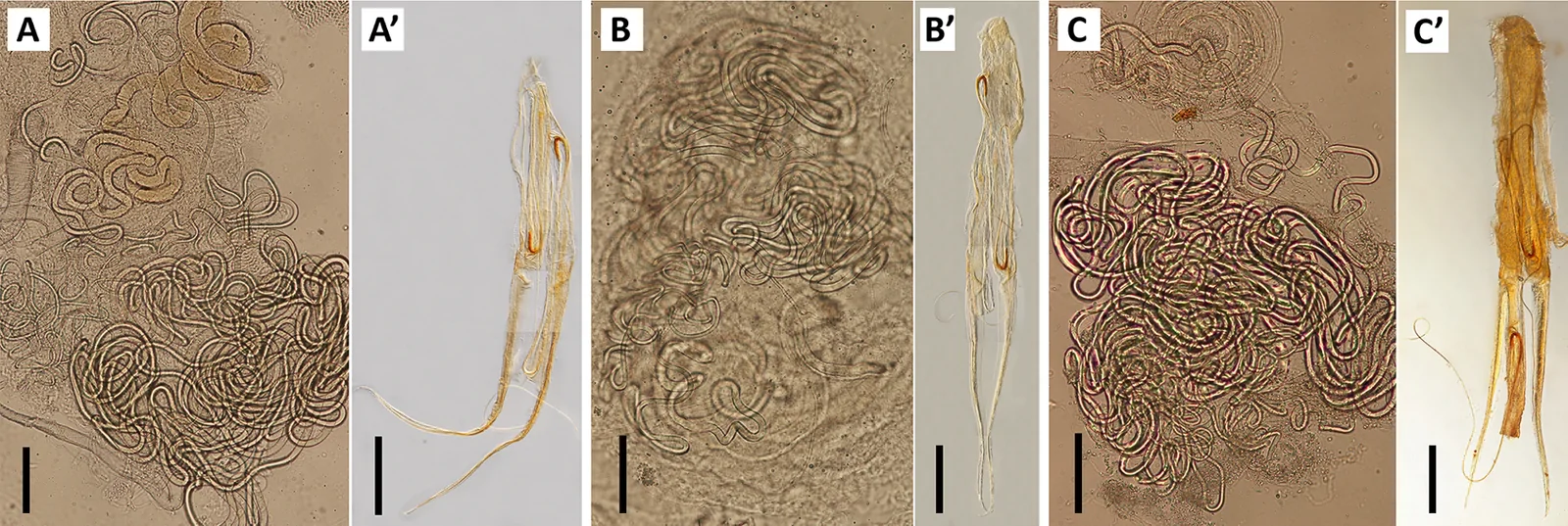
Spermatheca and male genitalia. **A, A’**. *Epilandex kawakamii* comb. nov.: 13vii23-5TW, 13vii23-4TW from Taiwan; **B, B’**. 25viii24-17LS, 25viii24-11LS from Laos; **C, C’**. *Epilandex peterseni*: 15iii24-4BN, 15iii24-1BN. Scale bars: 0.1 mm (spermatheca); 0.5 mm (male genitalia).

Figs 3, 5 A, A’, B, B’

Psalis kawakamii Shiraki, 1928: 5.
Carcinophora kawakamii : Sakai 1970: 77.
Carcinophora (Carcinophora) kawakamii : Sakai 1982: 30.
Epilandex acuminata Srivastava, 2003: 25, syn. nov.

##### Material examined.

***Holotype***. Taiwan • ♂; Arisan; 1912; T. Kawakami leg.; as *Psalis kawakamii* (Shiraki, 1928); examined from photographs; National Taiwan University Museum No. 1796.

##### Other material.

Taiwan • 6♂♂ 14♀♀; Nantou County, Ren-ai Township, Husui Forest Area, Frog Rock Tail; 24.093°N, 121.031°E; 13 July 2023; T. Yoshida leg; Light trap. Laos • 8♂♂ 4♀♀; Khammouan Province, Nakai District, Nakai-Nam Theun National Park; 17°45'44.4"N, 105°22'39.7"E; elev. 595 m; 25 August 2024; B. Chouangthavy leg; pitfall trap.

##### Measurements.

Holotype (male): body 10.0 mm; forceps 1.5 mm. Taiwan (male): body 9.9 mm; forceps 1.2 mm. Taiwan (female): body 10.1 mm; forceps 1.2 mm. Laos (male): body 9.0 mm; forceps 1.2 mm. Laos (female): body 8.5 mm; forceps 1.1 mm.

##### Redescription.

**Male**. Predominantly reddish-brown, with yellowish mouthparts on the head; apical antennal segments light yellowish; abdominal apex and forceps blackish-brown. Head slightly longer than broad; postfrontal and coronal sutures present; eyes prominent, slightly longer than head behind eyes. Antennae 17-segmented; first (basal) segment elongated, but slightly shorter than the distance between the antennal bases; second quadrate; third ~ 2× as long as wide. Remaining segments gradually lengthening and pyriform. Pronotum quadrate and all angles rounded; lateral margins subparallel; posterior margin rounded; fine median longitudinal furrow present. Tegmina well developed, longer, and wide than pronotum; some with well-developed hindwings (Fig. 3A, B) or very small only pointed tips visible from the dorsal (Fig. 3C). Sides of abdominal tergites VI–VIII are sharply pointed with clearly visible median longitudinal carina on each side (Fig. 3A”, B”, C”), but those VI are weaker (Fig. 3A”). Tergite V with trace of carina and a spine in some cases (Fig. 3B”); all tergites V–VIII are strongly carinated and taper backward like thorns in holotype (Fig. 3C”). Ultimate tergite broad, posterior margin concave. Forceps asymmetrical; left branch curved basally and apically, right branch curved more conspicuously apically. Penultimate sternite posteriorly subtruncate in the middle or faintly emarginated in the middle. Genitalia with very long and narrow parameres (Fig. 5A’, B’); basal piece (= central parameral plate of Steinmann 1986; = proparameres of Srivastava 1988) shorter almost half length of parameres, 3× as wide as base of parameres; virga also long, almost as long as parameres, with a well-sclerotized rod-like appendage at base.

**Female**. Similar to the male except the penultimate ventral sternite is rounded; the forceps are simple and symmetrical, with finely serrated inner margins. Spermatheca present as a very long, thin tube without a capsule at the distal end (Fig. 5A, B).

##### Distribution.

Taiwan (this study; Shiraki 1928, as *P. kawakamii*), China (Fujian: Chen and Ma 2004, as *C. kawakamii*); Hong Kong (Srivastava 2003a, as *Ep. acuminata*); Laos (this study, new record).

##### Remarks.

The body size of specimens from Taiwan and Laos agreed with that of the holotype of *Psalis kawakamii* (= *Carcinophora kawakamii*) (Fig. 3A–C), although they are slightly larger than the specimens from Hong Kong (Srivastava 2003a). Two other variations can be noted: first, Shiraki (1928) noted that the holotype has rudimentary hindwings, very small and pointed (Fig. 3C), but according to Srivastava (2003a)*Ep. acuminata* has well-developed hindwings, as all new specimens from Taiwan and Laos. Second, variation is also observed in the abdominal tergites: the new specimens have a well-developed median longitudinal carina on each of tergites VI–VIII (Fig. 3A”, B’’), with a trace of carina and a spine also on the tergum V only in the Laotian sample (Fig. 3B”). However, in the holotype of *Psalis kawakamii* (Fig. 3C”), each side of abdominal tergites V–VIII is strongly and sharply angled forming a well-developed carina.

#### Epilandex peterseni

Taxon classificationAnimaliaDermapteraAnisolabididae

Ramamurthi, 1967

7FD9AA7C-79A1-5890-BE11-8CCC9C2FFC85

Figs 4, 5 C, C’

##### Material examined.

***Holotype***. Philippines • ♂; Tawi Tawi, Tarawakan, north of Batu Batu; 22 October 1961; Noona Dan leg; Malaise traps; examined from photographs; Natural History Museum of Denmark, University of Copenhagen zmuc00028762. ***Allotype***. Philippines • ♀; Palawan, Mantalingajan Pinigisan; elev 600 ca; 24 September 1961; Noona Dan leg; Mercury-light; examined from photographs; Natural History Museum of Denmark, University of Copenhagen zmuc00028763.

##### Other material.

Indonesia • 2♂♂ 3♀♀; Borneo Island, West Kotawaringin Regency; 2.585198°S, 111.790678°E; 15 March 2024; S. Muharani and M. A. Pratama leg; pitfall trap.

##### Measurements.

Holotype (male): body 7 mm; forceps 1.2 mm. Allotype (female): body 8.1 mm; forceps 1.3 mm. Borneo (male): body 8.4 mm; forceps 1.3 mm. Borneo (female): body 8.3 mm; forceps 1.0 mm.

##### Redescription.

**Male**. Head black, rest of the body brownish, legs paler, antennal segments 12–17 whitish. Head, transverse, smooth and convex; sutures indistinct. Antennae 17-segmented; first (basal) segment elongated, but slightly shorter than the distance between the antennal bases; second quadrate; third ~ 2× as long as wide. Remaining segments gradually lengthening and pyriform. Pronotum, narrower than head anteriorly, slightly broadened posteriorly; lateral margins straight; posterior margin rounded; disc convex except for very narrow depressed areas laterally. Tegmina < 1.5× longer and wider than pronotum; hindwings well developed. Abdomen broadened posteriorly; VI–VIII laterally acute with distinct longitudinal carinae. Ultimate tergite broader than long, comparatively smooth, and weakly punctate; disc with a distinct median sulcus and a shallow caudal depression; posterior margin truncate medially and obliquely sinuate laterad. Forceps asymmetrical; inner margin entire; left branch abruptly narrowed at the apex, right branch gently arcuate. Penultimate sternite, broadly rounded. Genitalia (Fig. 5C’) similar to those of *Ep. kawakamii* comb. nov.

##### Female.

Agrees with male in general except the forceps are simple and symmetrical. Spermatheca, a very long, thin tube without a capsule at the distal end (Fig. 5C).

##### Distribution.

Philippines (Tawi Tawi: Ramamurthi 1967; Palawan: Ramamurthi 1967; Mindanao: Srivastava 1978); Bismarck Islands (New Britain and New Ireland: Srivastava 1976); Brunei (Kočárek 2021); Singapore (Kamimura et al. 2026); Indonesia (Borneo: this study, new record).

##### Remarks.

The specimens from Borneo examined in this study agrees well with the original description by Ramamurthi (1967) of *Epilandex peterseni*, as well as its type materials (Fig. 4A).

## Discussion

### Identification of *Epilandex* species

*Psalis kawakamii* Shiraki, 1928 was described based on a single male collected from Mount Arisan, Taiwan (Shiraki 1928). Because the generic name *Psalis* Audinet-Serville, 1831 is a junior homonym and is therefore invalid in Dermaptera, several species formerly placed in this genus were transferred to *Carcinophora* Scudder, 1876. However, *P. kawakamii* was neglected for many years until Sakai (1970) transferred it to *Carcinophora* (Sakai 1970; Steinmann 1984, 1989a, 1989b).

The holotype of *Ep. kawakamii* comb. nov. and the additional Taiwanese specimens have slightly larger body sizes (9–10 mm including forceps) than the Laotian specimens (less than 9 mm). According to Srivastava (2003a), the specimens from Hong Kong (as *Ep. acuminata*) have a smaller body size of less than 9 mm in both sexes.

The Taiwanese and Laotian specimens obtained in this study generally agree with the original description by Shiraki (1928) and the morphology of the holotype (Fig. 3C), except for the following differences: all of our specimens from Taiwan and Laos (males and females), as well as the specimen from Hong Kong (as *Ep. acuminata*), have well-developed hindwings (Srivastava 2003a), whereas the holotype of *Psalis kawakamii* (now *Ep. kawakamii*) (Fig. 3C) possesses very small and pointed hindwings (Shiraki 1928). Previous studies have reported intraspecific variation in hindwing development for several *Epilandex* species such as *Ep. solomonensis* and *Ep. burri* (Ramamurthi 1967; Sakai 1987; Steinmann 1989a, 1989b; Srivastava 2003b). Accordingly, the development of hindwings cannot be used as a reliable diagnostic character (Kočárek 2021).

Similarly, the number of abdominal tergites with carinae was also variable among the specimens examined in this study. According to Srivastava (2003a), *Ep. acuminata* exhibits these characteristics on segments VI and VII, as sharply angled and pointed carinae. Shiraki (1928) described *Psalis kawakamii* (now *Ep. kawakamii*), in which the sides of abdominal tergites VI–IX (apparently a misinterpretation of V–VIII) are strongly and sharply angled and taper backward like thorns. This description was confirmed by Sakai (1982) and our own observations of the holotype (Fig. 3C’’), indicating that the carination is more pronounced in this specimen. Variation in the development of the abdominal carinae is also known in *Ep. burri*. The original description stated that the sides of the abdominal segments IV–IX possess a carina on each side (Borelli 1921), whereas Srivastava (1982, 1988) reported that Indian specimens lack these oblique carinae. Brindle (1970) and Kamimura et al. (2026) also reported variation in carina development in *Ep. solomonensis* and *Ep. peterseni*, respectively. Therefore, the degree of carinal development on the abdominal tergites alone is insufficient as a diagnostic character for distinguishing *Epilandex* species.

Nevertheless, considering the very low level of genetic differentiation detected in the DNA barcoding region (up to 1.56%) compared to the 2.0% threshold often used as a standard for species boundaries (Cognato 2006), we tentatively conclude that all the *Epilandex* specimens recorded from the subtropical regions of Laos, China (including Hong Kong), and Taiwan belong to a single species. The limited availability of specimens and the high similarity in external morphology among *Epilandex* species have resulted in poorly defined diagnostic characters for species identification (Kočárek 2021). Our findings indicate that the shape of the penultimate sternite (rounded in the Bornean clade, but emarginate in the Taiwan + Laos clade) is strongly correlated with the genetic divergence revealed in the phylogenetic analysis.

The spermatheca of *Epilandex* species is described here for the first time as a very long, thin, and blind duct lacking a capsule at the distal end. This simple, tubular type of spermatheca has been reported for many anisolabidid species except for *Platylabia major* Dohrn, 1867 (Platylabiinae) (Kamimura et al. 2016).

### Placement of *Epilandex* in Anisolabididae

Our molecular analysis (Fig. 1A) demonstrates that *Epilandex* merits recognition at the subfamily rank. In addition to the distinctive male genitalia with notably elongate parameres (Fig. 5), the unique metasternal morphology separates *Epilandex* from other genera of Anisolabidinae. However, due to the misidentification by Burr (1915) and subsequent taxonomic reassignments outlined above (see Taxonomy and nomenclature), no appropriate name is available for this taxon. To accommodate the morphological concept originally intended for Landicinae Burr, 1915, we propose the new subfamily name Epilandicinae nom. nov. with the modified definition provided above. Srivastava (1999) provided the primary framework for distinguishing subfamilies within Anisolabididae based on body shape, the structure of thoracic tergites and sternites, and male genitalia. In this study, we revised that key by excluding Gonolabininae Popham & Brindle, 1966 following Kamimura (2025).

We observed that the metasternum of *Epilandex* narrows posteriorly, with a distinct constriction at the base of the hind legs. This character was consistently found in both sexes, and thus serves as a diagnostic character of this genus. Our *Epilandex* specimens from Borneo were collected in a conservation forest within oil palm plantations (PT Surya Sawit Sejati), which harbors a high diversity of insects. On the other hand, the specimens from Taiwan and Laos were obtained using light traps. Many *Epilandex* specimens examined in previous studies were also captured using light, Malaise, or flight-intercept traps (Kočárek 2021; Kamimura et al. 2026). These lines of evidence suggest that members of this genus predominantly inhabit arboreal habitats and consequently fly frequently. This pattern implies a possible association between metasternal morphology and climbing behavior. We hypothesize that the posterior narrowing of the metasternum facilitates greater mobility of the hind legs, potentially enhancing locomotion on vertical substrates. This contrasts with other members of Anisolabidinae*sensu stricto*, which are predominantly terrestrial and lack this modification.

## Supplementary Material

XML Treatment for Epilandicinae

XML Treatment for Epilandex

XML Treatment for Epilandex kawakamii

XML Treatment for Epilandex peterseni

## Additional information

### Artificial Intelligence (AI) use

The authors accept full responsibility for the content of the manuscript, including the disclosure of any use of AI.

Regarding the use of AI in the preparation of this manuscript, the authors declare the following: Gemini.

**Used for**: Language, style and writing.

During the preparation of this manuscript, the authors used Google Gemini solely to improve the English language and readability. After using the tool, the authors reviewed and edited the content as needed and take full responsibility for the final publication.

### Author ORCIDs

M. Gusti https://orcid.org/0000-0001-6936-4089

Y. Kamimura https://orcid.org/0000-0003-2882-491X

B. Chouangthavy https://orcid.org/0000-0003-1715-6434

C.-F. Lee https://orcid.org/0000-0003-1996-0557

M. Silmi https://orcid.org/0009-0006-6527-8072

K. Eguchi https://orcid.org/0000-0002-1054-1295

### Data availability

All of the data that support the findings of this study are available in the main text or Supplementary Information.

## Supplementary materials

Supplementary material 1 Phylogenetic tree Data type png Explanation note **fig. S1**. Phylogenetic tree of *Epilandex* based on mitochondrial COI region using ML method with bootstrap value > 50 is show on the node. https://binary.pensoft.net/file/1802840This dataset is made available under the Open Database License (http://opendatacommons.org/licenses/odbl/1.0/). The Open Database License (ODbL) is a license agreement intended to allow users to freely share, modify, and use this Dataset while maintaining this same freedom for others, provided that the original source and author(s) are credited. Meliana Gusti, Yoshitaka Kamimura, Bounsanong Chouangthavy, Chi-Feng Lee, Muhammad Silmi, Ali Amran I, Katsuyuki Eguchi
